# Beta Oscillatory Dynamics in the Prefrontal and Superior Temporal Cortices Predict Spatial Working Memory Performance

**DOI:** 10.1038/s41598-018-26863-x

**Published:** 2018-05-31

**Authors:** Amy L. Proskovec, Alex I. Wiesman, Elizabeth Heinrichs-Graham, Tony W. Wilson

**Affiliations:** 1 Department of Psychology, University of Nebraska - Omaha, Omaha, NE USA; 2 Center for Magnetoencephalography, University of Nebraska Medical Center (UNMC), Omaha, NE USA; 3 Department of Neurological Sciences, UNMC, Omaha, NE USA

## Abstract

The oscillatory dynamics serving spatial working memory (SWM), and how such dynamics relate to performance, are poorly understood. To address these topics, the present study recruited 22 healthy adults to perform a SWM task during magnetoencephalography (MEG). The resulting MEG data were transformed into the time-frequency domain, and significant oscillatory responses were imaged using a beamformer. Voxel time series data were extracted from the cluster peaks to quantify the dynamics, while whole-brain partial correlation maps were computed to identify regions where oscillatory strength varied with accuracy on the SWM task. The results indicated transient theta oscillations in spatially distinct subregions of the prefrontal cortices at the onset of encoding and maintenance, which may underlie selection of goal-relevant information. Additionally, strong and persistent decreases in alpha and beta oscillations were observed throughout encoding and maintenance in parietal, temporal, and occipital regions, which could serve sustained attention and maintenance processes during SWM performance. The neuro-behavioral correlations revealed that beta activity within left dorsolateral prefrontal control regions and bilateral superior temporal integration regions was negatively correlated with SWM accuracy. Notably, this is the first study to employ a whole-brain approach to significantly link neural oscillations to behavioral performance in the context of SWM.

## Introduction

Spatial working memory (SWM) supports the temporary maintenance and manipulation of spatial information to be used towards concurrent processing, and is vital to healthy daily functioning. Like other forms of working memory, it can be broken down into three phases. Encoding encompasses the loading of information into memory, while maintenance refers to the active retention of that information. Finally, retrieval involves the recovery of the information to be used towards a specific cognitive operation.

Previous neuroimaging studies have shown that a variety of cortical regions within the frontal, temporal, parietal, and occipital cortices are recruited during SWM performance^1–8^. Some of these regions, including bilateral inferior frontal gyri, dorsolateral prefrontal cortices, insula, superior parietal lobules, intraparietal sulci, and pre-supplementary motor area are believed to be part of a core working memory (WM) network, which is activated across all subtypes of WM tasks (e.g., verbal, spatial, object)^1,3–5,9,10^. However, specific regions within and outside the core network also appear to serve particular types of WM operations. For example, stronger activation in the superior parietal lobules and intraparietal sulci (both core regions) during SWM relative to other types of WM has been reported, and, unlike other types, SWM processing appears to uniquely recruit the bilateral superior frontal gyri and right inferior parietal lobule^3–7,10–12^.

These anatomical findings have been confirmed and expanded by several large-scale meta-analyses examining the brain regions serving SWM performance^1,3–5,12^. However, these meta-analyses included only functional magnetic resonance imaging (fMRI) and positron-emission tomography (PET) results, and less is known about the oscillatory dynamics underlying SWM performance. The studies that have investigated oscillatory activity serving SWM have found similar brain regions despite utilizing a variety of tasks. For example, several studies have reported alpha oscillatory responses in frontal, parietal, and occipital regions during SWM performance. However, the results of these studies have been inconsistent, with some reporting increased^13–16^ and others finding decreased alpha activity within the same regions^16–21^. Other studies have reported increased alpha activity as WM load increased within SWM-related regions^15,17^, while the opposite load-related effects have also been observed^13,18,21–23^. Beyond the alpha findings, multiple SWM studies have reported beta oscillations in posterior areas^14,18,21^, increased theta activity in frontal and parieto-occipital cortices^20,22,23^, and increased gamma oscillations in frontal, temporal, and parietal regions^14,15,24^. Several studies have also found both increased and decreased gamma activity in occipital regions during SWM^14,18,21^.

While the aforementioned studies have provided critical insight into the oscillatory dynamics serving SWM, a few points become apparent when considering this work. First, nine of the studies restricted their analyses to the maintenance phase of SWM performance, two utilized tasks in which encoding, maintenance, and retrieval processes occurred in parallel, and the final study did not attempt to identify the neural origin of oscillatory responses. Thus, the spatiotemporal dynamics underlying the distinct phases of SWM remain poorly understood. Second, the at times conflicting results and wide array of SWM tasks utilized render a direct comparison between, and conclusive interpretation across studies difficult. Third, only one study investigated the relationship between oscillatory responses and SWM performance. This is surprising given that a hand-full of fMRI experiments have revealed that neural activity within bilateral prefrontal cortices, frontal eye fields, and posterior parietal cortices is tied to better SWM performance, with the dorsolateral prefrontal cortex being most often cited^25–30^. Additionally, the study that did probe oscillation-performance relationships constricted the analyses to gamma activity within two regions of interest, demonstrating that gamma activity within the left medial prefrontal cortex could be used to decode the number of behaviorally relevant items held in SWM with a 59% probability of correct assignment^15^. While this finding was an important contribution to the field, much remains to be explored. In particular, a whole-brain analysis approach could offer crucial insight into the oscillatory correlates of SWM performance.

In the present study, we sought to fill the aforementioned gaps in the literature and characterize the oscillatory dynamics serving SWM encoding and maintenance processes, including being the first study to investigate the relationship between oscillatory activity and SWM performance via whole-brain analyses. To this end, we utilized the spatiotemporal precision of magnetoencephalography (MEG) and a visual SWM task. Our hypotheses were two-fold. First, given the established functional specializations of distinct prefrontal regions and the prior reports of increased frontal theta during WM performance^20,22,23,31–33^, we predicted increased theta activity in prefrontal regions that would be differentially recruited during SWM encoding and maintenance processes. In contrast, we hypothesized persistent decreases in alpha and beta activity in posterior parietal and occipital regions throughout SWM encoding and maintenance, as decreased alpha and/or beta activity has been linked to active engagement in ongoing cognitive processing^18,34,35^, and given the involvement of posterior parietal and occipital regions in spatial attention and mapping the spatial environment^36–39^. Our second set of hypotheses pertained to the relationships between neurophysiological and behavioral data. Provided the consistent link between dorsolateral prefrontal cortex activity and SWM performance in the fMRI literature^25–27,30^, we hypothesized that oscillatory activity within the dorsolateral prefrontal cortex would similarly be related to SWM performance.

## Results

### Behavioral Analysis

**Figure 1:**
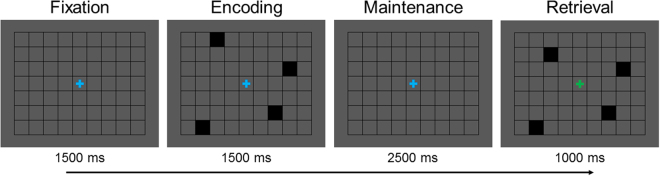
Spatial working memory task.

Twenty-two healthy adults (mean age: 26.05) were recruited to perform a visual SWM task during MEG. During task performance, all participants were seated in a nonmagnetic chair within a magnetically-shielded room and told to fixate on stimuli presented centrally. Briefly, a trial consisted of the presentation of an empty 7 × 9 grid for 1500 ms, four black squares displayed within the grid for 1500 ms (encoding), an empty grid for 2500 ms (maintenance), and a probe of four black squares presented within the grid for 1000 ms (retrieval) during which participants responded as to whether the squares were in the same location relative to the encoding stimulus (Fig. 1). Each trial lasted 6500 ms, and each participant completed approximately 128 trials. All 22 participants were able to complete the task, with a mean accuracy of 81.6% (*SD* = 7.3%) and an average latency of 891.3 ms (*SD* = 146.5 ms).

### Sensor-Level Analysis

**Figure 2:**
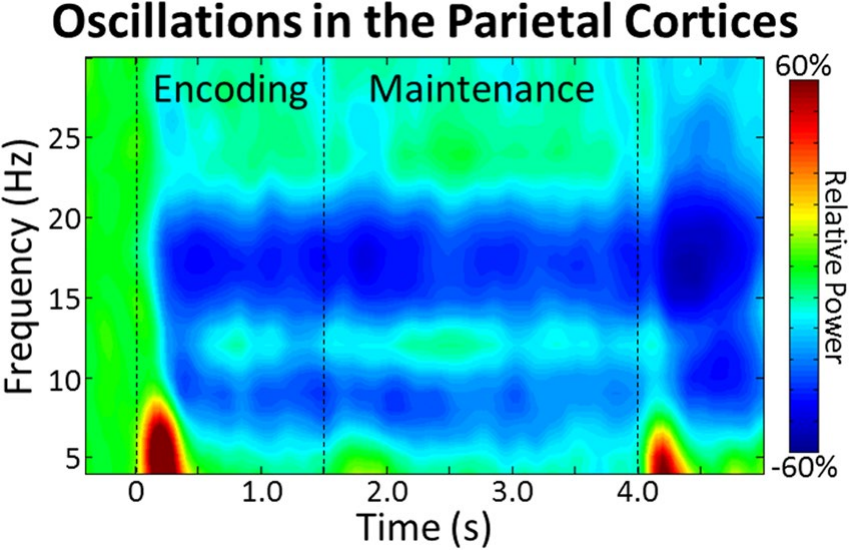
Time-frequency spectrogram with time (s) shown on the x-axis and frequency (Hz) denoted on the y-axis. Percent power change was computed for each time-frequency bin relative to the respective bin’s baseline power (−0.4 to 0.0 s). The color legend is displayed to the right. Data represent a grand-averaged peak sensor located near the parietal cortices (the same sensor was used in all participants). A strong and transient increase in theta activity was observed shortly following encoding onset. Slightly later during encoding strong decreases in alpha and beta activity were found, which were sustained throughout the remainder of encoding and maintenance.

The continuous magnetic time series was divided into epochs of 5500 ms duration, with the onset of the encoding stimulus being defined as 0 ms, the onset of maintenance occurring at 1500 ms, and retrieval onset occurring at 4000 ms. To determine time-frequency bins containing oscillatory responses of interest, artifact-free sensor-level data was transformed into the time-frequency domain using complex demodulation, and normalized relative to a baseline period (i.e., −400 to 0 ms). Statistical analysis of the resulting time-frequency spectrograms revealed significant clusters of theta (4–8 Hz), alpha (8–11 Hz), and beta (15–20 Hz) oscillatory activity in gradiometers near temporal, parietal, and occipital cortices across all participants (Fig. 2; *p* < 0.05, corrected). Shortly after the onset of the encoding stimulus (i.e., 0 ms), significant increases in theta activity were observed beginning at 100 ms, which tapered off at about 350 ms (*p* < 0.05, corrected). The alpha and beta responses were much more sustained, with significant decreases in both bands beginning about 400 ms after the onset of the encoding stimulus, and persisting throughout the remainder of the encoding and maintenance periods (*p* < 0.05, corrected). Thus, the time-frequency windows which were beamformed were: 4 to 8 Hz from 100 to 350 ms, 8 to 11 Hz from 400 to 4000 ms, and 15 to 20 Hz from 400 to 4000 ms.

### Beamformer and Virtual Sensor Analyses

**Figure 3:**
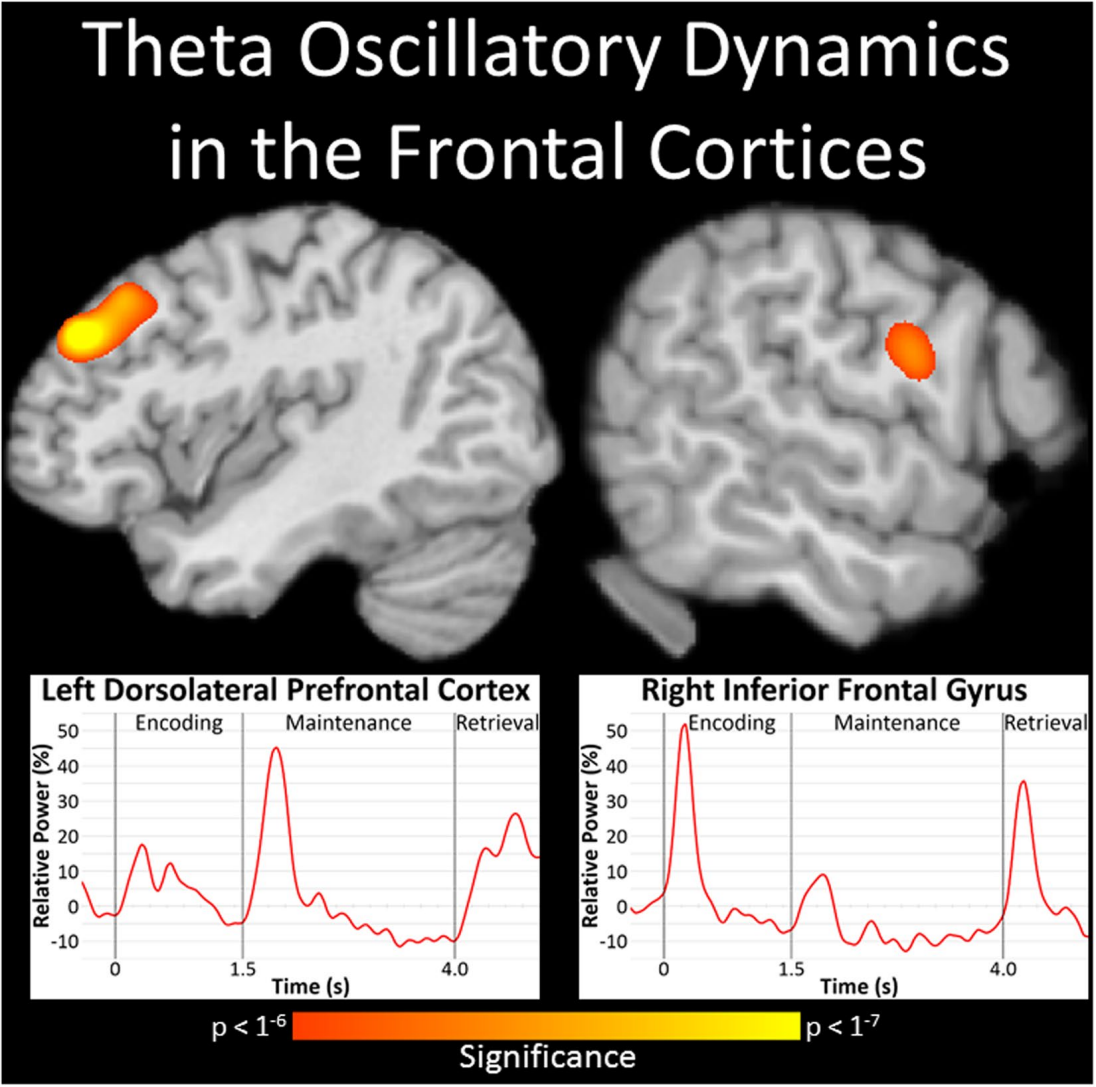
Theta oscillations during spatial working memory performance. Significant increases in theta (4–8 Hz) activity were observed in the left dorsolateral prefrontal cortex (DLPFC; top left) and right inferior frontal gyrus (IFG; top right) shortly after the onset of the encoding stimulus (*p* < 1^−6^, cluster-corrected). Time courses of theta activity from the peak voxel of each region revealed a transient increase in theta activity following the onset of the encoding grid, with a more substantial increase seen in the right IFG (bottom right). A transient increase in theta activity was also found at the beginning of the maintenance phase, but in this case the response was much stronger in the left DLPFC (bottom left).

Imaging of the oscillatory responses identified through the sensor-level analyses revealed strong increases in theta activity in the left dorsolateral prefrontal cortex and right inferior frontal gyrus (Fig. 3; *p* < 1^−6^, cluster-corrected). To characterize the time course of activity in these regions, we extracted virtual sensors from the peak voxel in each cluster, and plotted their relative power as a function of time. The time series of the left dorsolateral prefrontal cortex revealed transient increases in theta activity shortly after the onset of both the encoding and maintenance phases, with that following the onset of maintenance being markedly stronger. Theta activity in the right inferior frontal gyrus followed a similar pattern, but the more substantial increase was observed following the onset of the encoding stimulus.

**Figure 4:**
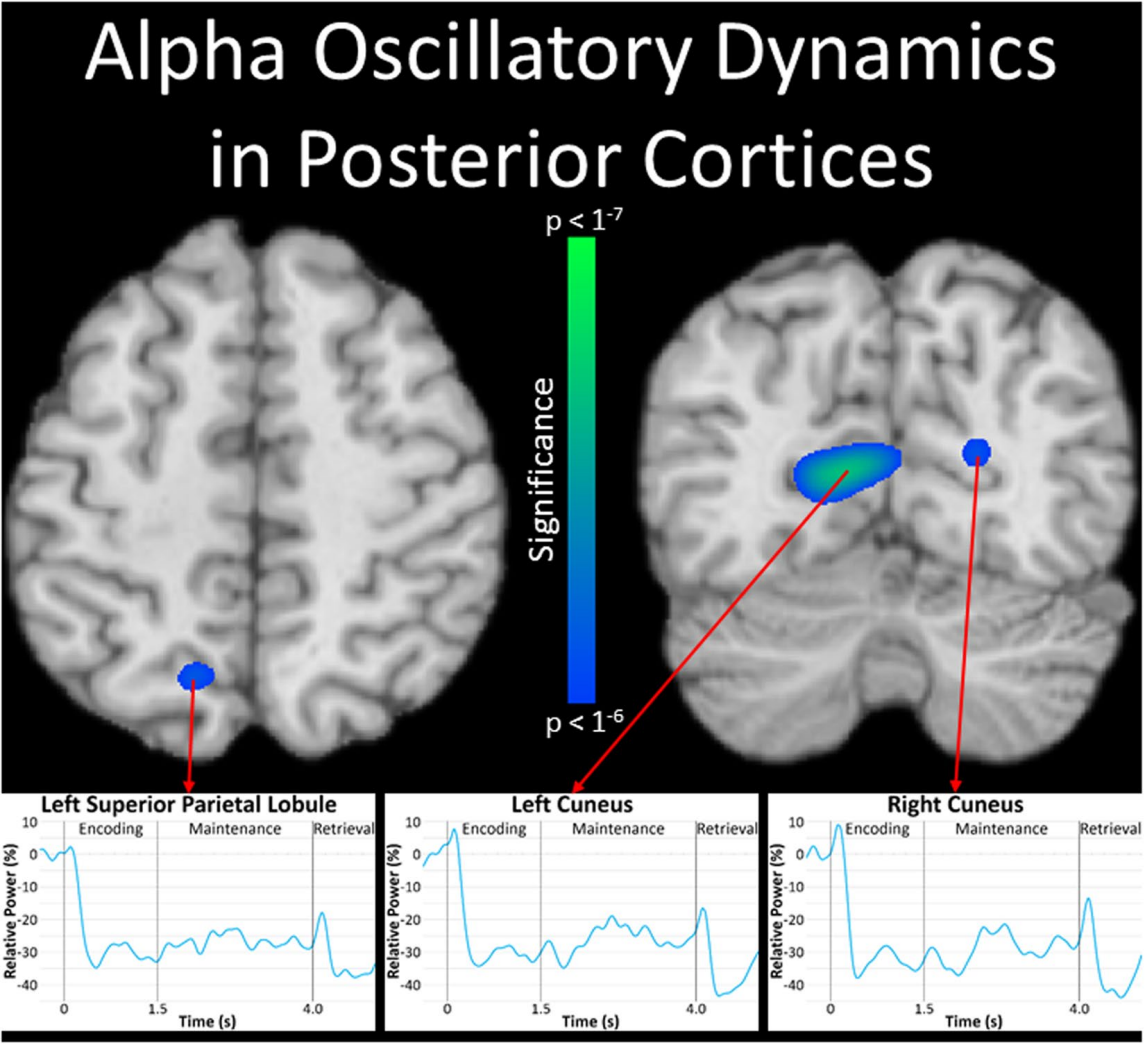
Alpha oscillations during spatial working memory performance. Significant decreases in alpha (8–11 Hz) activity were observed in the left superior parietal lobule (top left) and bilateral cuneus (top right) throughout the majority of the encoding and maintenance periods (*p* < 1^−6^, cluster-corrected). The alpha time series from the peak voxel of each region revealed strong decreases following the onset of the encoding stimulus across all three regions (see bottom panels), and these decreases persisted throughout the remainder of encoding and maintenance processes.

**Figure 5:**
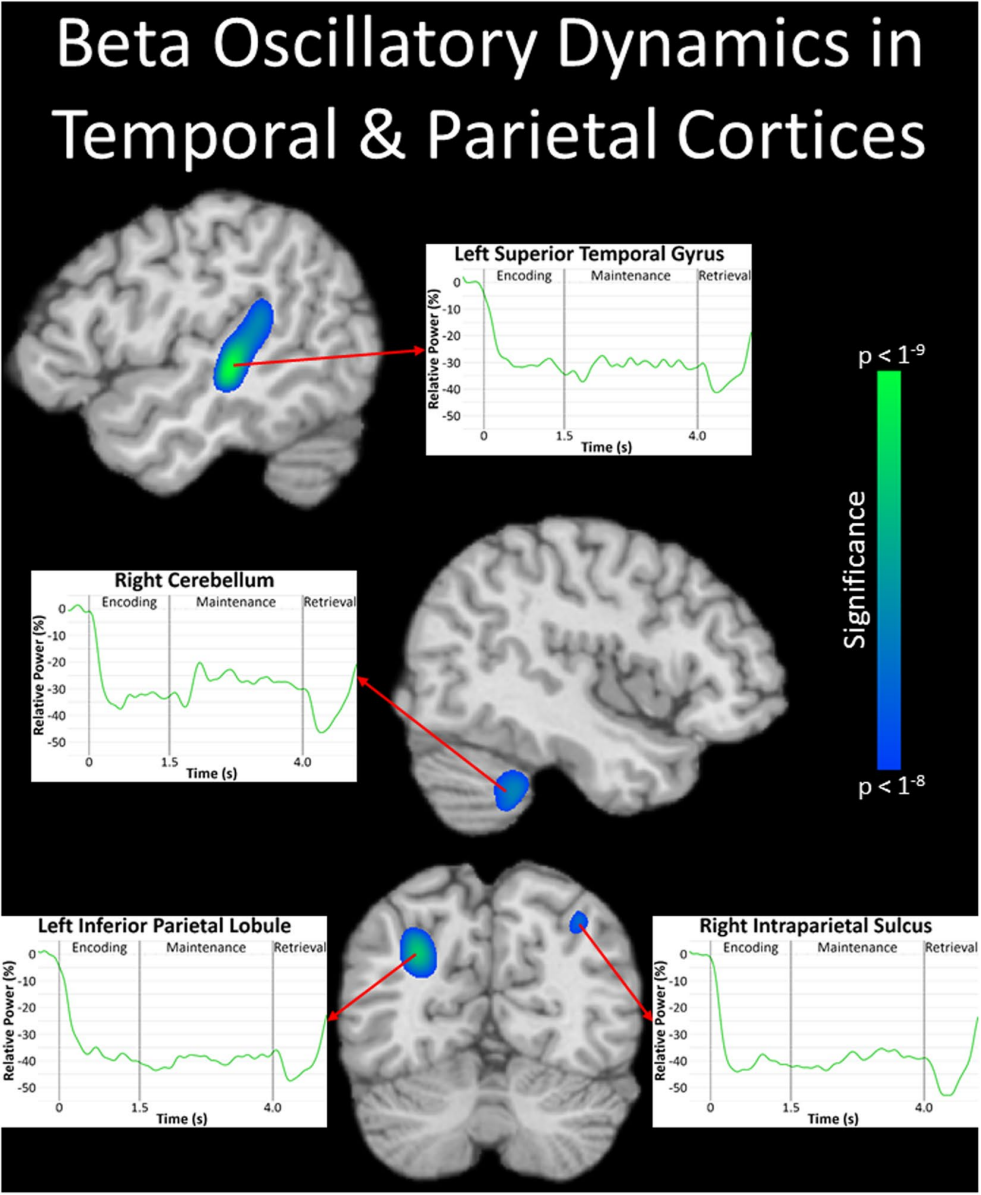
Beta oscillations during spatial working memory performance. Significant decreases in beta (15–20 Hz) activity were observed in the left superior temporal gyrus (top left), right cerebellum (middle central), left inferior parietal lobule, and right intraparietal sulcus (bottom central) throughout the majority of encoding and maintenance (*p* < 1^−8^, cluster-corrected). Time courses of beta activity from the peak voxel of each region indicated large decreases immediately following the onset of the encoding grid, which were clearly sustained throughout the remainder of encoding and maintenance.

In contrast, significant decreases in alpha and beta activity were observed in more posterior regions including the left superior parietal lobule and bilateral cuneus for alpha activity (Fig. 4), and the left superior temporal gyrus, left inferior parietal lobule, right intraparietal sulcus, and right cerebellum for beta activity (Fig. 5). Inspection of the time series revealed sharp decreases in alpha and beta activity shortly after the onset of the encoding stimulus across their respective regions, which were largely sustained throughout the remainder of encoding and the entirety of maintenance. Of note, we also conducted the same virtual sensor time series analyses with the evoked activity removed for the theta and alpha peaks, and found very similar results for these regions. Essentially, the resulting temporal profiles of oscillatory dynamics were nearly identical across the two approaches, albeit the amplitude of responses varied in some regions (see Supplementary Figs S1 and S2). Thus, the responses observed in the time series analyses were above and beyond the influence of evoked responses.

### SWM Performance Correlation Maps

**Figure 6:**
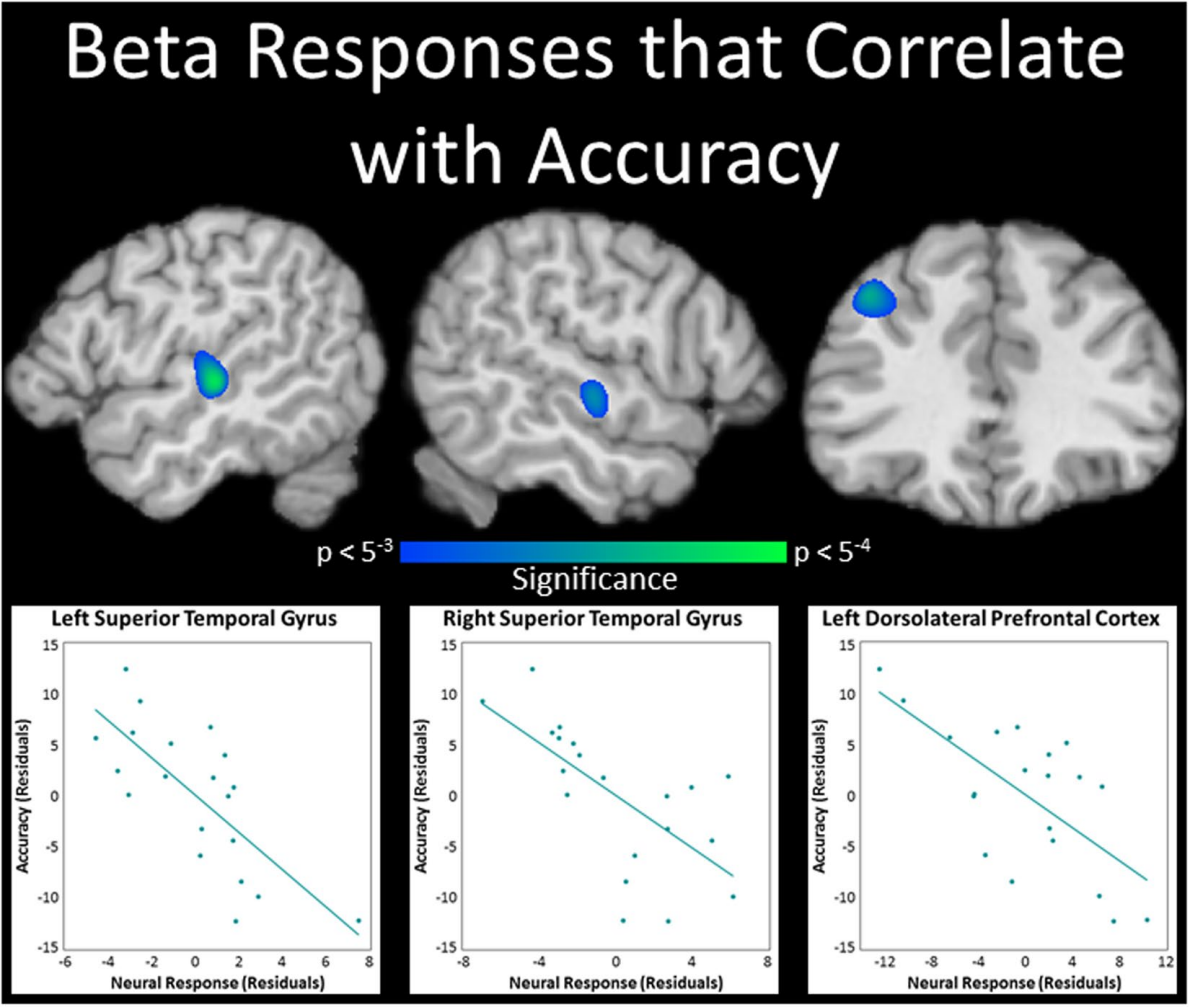
Beta oscillations related to spatial working memory (SWM) accuracy. Beta (15–20 Hz) activity within the left superior temporal gyrus (STG; top left), right STG (top central), and left dorsolateral prefrontal cortex (top right) was negatively correlated with accuracy on the SWM task, when partialling out reaction time (*p* < 0.005, cluster-corrected). Scatterplots representing these relationships are presented below utilizing the data from the peak voxels of the whole-brain partial correlation maps.

To identify oscillatory responses that were directly tied to SWM performance, whole-brain correlation maps were computed using images from each MEG time-frequency response and accuracy. This revealed that beta oscillatory activity within the left dorsolateral prefrontal cortex and bilateral superior temporal gyri was negatively correlated with accuracy on the SWM task, when partialling out reaction time (Fig. 6; *p* < 0.005, cluster-corrected). That is, as beta activity decreased within these regions, SWM performance tended to increase. No significant correlations were detected between theta activity and accuracy nor alpha activity and accuracy.

## Discussion

In this investigation, we examined the spatiotemporal dynamics of SWM processing in healthy adults using MEG, and tested whether such oscillatory responses were directly related to SWM performance. Our results indicated strong increases in theta activity in the left dorsolateral prefrontal cortex and right inferior frontal gyrus shortly after the onset of the encoding phase, with the local time series revealing transient increases in theta shortly after the initiation of maintenance in both regions, as well. We also found large decreases in alpha activity in the left superior parietal lobule and bilateral cuneus, and strong decreases in beta activity in the left superior temporal gyrus, left inferior parietal lobule, right intraparietal sulcus, and right cerebellum. In contrast to the transient nature of the theta responses, the alpha and beta decreases were sustained across the majority of the encoding and maintenance phases of this SWM task. Finally, stronger decreases in beta activity within the left dorsolateral prefrontal cortex and bilateral superior temporal gyri were related to greater SWM accuracy. The implications of these results are discussed in turn below.

As previously mentioned, substantial evidence supports the existence of a core network of brain regions that serve WM performance across multiple types of stimuli^3,5^. Not surprisingly, nodes within this core network are believed to oversee and/or coordinate WM processing at large, and prefrontal regions are integral to this overarching executive system^33,40–46^. Particularly, evidence suggests that areas of the ventrolateral prefrontal cortex (e.g., the inferior frontal gyrus) are involved in the selection of task-relevant information during WM performance^31,32^, and prior neurophysiological research has suggested that theta oscillations may play a prominent role in such selection and integration during WM processing^46^. Consistent with this framework, we observed the strongest inferior frontal theta response following the onset of the encoding grid, when the initial selection and integration of the spatial locations would logically occur.

Beyond the ventrolateral prefrontal cortices, a wealth of research has also implicated the dorsolateral prefrontal cortices in WM performance, tying them to higher-order maintenance and manipulation processes^31–33^, as well as SWM accuracy^25–30^. Our data also supports a central role for the dorsolateral prefrontal cortices in SWM processes. First, our results demonstrate that the strongest increase in dorsolateral prefrontal theta activity followed the onset of the maintenance phase, and previous neurophysiological research has suggested that such activity may help coordinate activity in more posterior, task-relevant regions during WM maintenance^43,46,47^. Second, greater decreases in beta activity within the dorsolateral prefrontal cortex were associated with better SWM performance. That is, similar to alpha activity, decreased beta within a neural region is generally believed to reflect the active engagement of that region in ongoing processing^18,34,35,48^. This position has been supported by studies utilizing simultaneous EEG-fMRI, which have demonstrated negative correlations between low frequency (i.e., alpha/beta) oscillatory activity and the fMRI BOLD signal during cognitive tasks^49–53^. In other words, these studies found that greater fMRI activation was associated with reduced EEG alpha/beta power in roughly the same region. Thus, our results align well with the previous fMRI research linking stronger dorsolateral prefrontal cortex activations to better SWM performance^25–30^, and also compliment the body of non-human primate research demonstrating that persistent firing in prefrontal cortical neurons during SWM encoding and maintenance predicts accurate performance^42,54^. Amalgamating our findings, the increased theta activity in the inferior frontal gyrus may reflect the selection of task-relevant information early during encoding, while the oscillatory mechanisms observed in the dorsolateral prefrontal cortex may reflect top-down executive control processes important for accurate SWM performance.

While the prefrontal cortices have been implicated in higher-order executive roles across various WM tasks, previous research has shown that regions within the posterior parietal cortex are more intimately tied to SWM. Critically, the existence of spatial maps within the human intraparietal sulci and the non-human primate homologue (i.e., lateral intraparietal areas) has been widely documented, and such maps are believed to have a major role in holding sensory representations of specific locations during SWM performance^2,38,39,55–58^. In a similar vein, these intraparietal regions, along with the superior parietal lobules and occipital regions, are included in the well-known dorsal-visual stream^11^. This set of regions is commonly referred to as the “where” pathway, as it is closely tied to the extraction and processing of location information from the visual space at large^11,59^. Moreover, these superior parietal regions are implicated in the top-down control of spatial attention, and are believed to enhance the processing of sensory information pertinent to the current behavioral goal^60–63^. Previous studies have shown that alpha and beta oscillatory activity has a central role in such processes. For example, sustained decreases in occipital alpha have been tied to visual attention, and further have been shown to be under top-down modulation during visuospatial tasks^36,37,64^. Consistent with such occipital responses, decreases in alpha and beta activity within posterior parietal regions have been found in multiple studies of visuospatial attention^36,37,65–71^, and such decreases could be the result of top-down control. Additionally, decreased beta activity within occipital and parietal regions has been implicated in feature-specific processing during WM, with decreases in the dorsal visual stream tied to the retention of spatial features^21^. Taken together, the sustained beta decreases that we observed within the intraparietal sulcus may reflect the active maintenance of the to-be-remembered visuospatial locations, with the sustained alpha decreases found in superior parietal and occipital regions being a consequence of the sustained attention to hold multiple visual locations during the maintenance phase.

In addition to the commonly cited prefrontal, parietal, and occipital involvement in SWM, our data indicates a somewhat surprising and notable role of the superior temporal gyri in the accurate performance of our SWM task. While arguably not given as much attention in the SWM literature, activity in these regions has been widely reported in visuospatial attention and WM tasks^14,29,63,72–74^. In fact, decreased beta and gamma activity within superior temporal regions has been observed during visual WM maintenance when using a similar change-detection task of colored squares^75^. Park *et al*.^14^ also used a task similar to ours and found that the right superior temporal gyrus had increased gamma oscillatory activity during SWM maintenance, and that gamma within this region correlated with increased alpha activity in frontal, temporal, and cerebellar regions, and increased beta activity in occipital regions^14^. Of additional interest, a study in non-human primates demonstrated greater beta phase synchrony within left superior temporal regions during the performance of correct relative to incorrect trials in a visual WM task^76^, and a similar accuracy effect was found in an overlapping temporal region in a human fMRI study on SWM^29^. Previous studies have linked the superior temporal gyri with the processing of relations between simultaneously presented stimuli^14,63,73,74^, and considering our task involved the encoding and maintenance of multiple locations, the sustained beta decreases observed here could reflect a similar relational mechanism. If this interpretation is correct, it may be the higher-order integration and maintenance operations, rather than the sensory representations of the independent locations themselves, that are intimately tied to accurate performance on the SWM task utilized here.

Finally, we also observed decreased beta activity within the cerebellum during SWM encoding and maintenance processes. These data are in line with previous MEG investigations of WM, which similarly reported decreases in alpha/beta cerebellar activity^72,77–79^, and compliment fMRI and PET research that has demonstrated the recruitment of similar cerebellar regions during WM encoding and maintenance processes^80–84^. Evidence suggests that the cerebellum is involved in the extraction of relevant information from the environment during visuospatial processes^85^, and may support the maintenance of such information during WM performance^80,84^.

While our results offer novel insight into the temporal dynamics associated with accurate SWM performance, our study was not without limitations. For example, by design, our study was restricted to healthy younger adults, and consequently we cannot comment on the WM deficits that are often associated with aging^86^. Age-related differences in the neural mechanisms serving WM have been widely reported^78,87–91^, and future investigations focusing on SWM should examine the effects of age on the oscillatory dynamics serving SWM. Another key parameter is biological sex, where differences in WM have been previously reported^92^, and future studies focusing on oscillations in this area are certainly needed. Finally, our study focused on visual SWM, but SWM can be assessed in the auditory domain, as well. While fMRI studies have reported considerable overlap between the neural regions recruited during auditory SWM and those engaged by visual SWM, evidence suggests that inferior frontal and frontal pole regions are recruited more heavily during auditory SWM^93–95^. Additionally, a handful of oscillatory studies have reported increased parieto-occipital alpha activity, as well as increased gamma activity in parietal and frontal regions during auditory SWM, with positive correlations observed between parietal gamma and behavioral performance^96–99^. As such, investigating the similarities and differences in the oscillatory dynamics serving visual and auditory SWM would be an interesting future direction.

In conclusion, this study utilized MEG to identify the oscillatory dynamics underlying SWM encoding and maintenance processes, and was the first to investigate the relationship between oscillatory responses and visual SWM performance by utilizing a whole-brain approach. Our results showed strong transient increases in frontal theta that were much stronger at the beginning of the encoding period in some regions (right inferior frontal) and at the beginning of the maintenance period in other regions (left prefrontal cortex). We propose that such theta activity may reflect the selection and integration of visual information relevant to the current behavioral goal. In contrast, prolonged decreases in alpha and beta activity in parietal, occipital, and temporal cortices may be more closely involved in sustained visuospatial attention, and the active maintenance of spatial location representations that are necessary for performance in this SWM task. Finally, prefrontal executive control systems and superior temporal spatial relation integration mechanisms, reflected through decreases in beta activity, may be of particular importance for SWM accuracy.

## Methods and Materials

### Subject Selection

We studied 22 healthy adults (11 females; mean age: 26.05, *SD*: 4.02, range: 21–35) who were recruited from the local community. Exclusionary criteria included any medical illness affecting central nervous system function, neurological or psychiatric disorder, history of head trauma, current substance abuse, and the MEG Laboratory’s standard exclusion criteria (e.g., dental braces, metal implants, and/or any type of ferromagnetic implanted material). After a complete description of the study, written informed consent was obtained from participants following the guidelines of the University of Nebraska Medical Center’s Institutional Review Board, which reviewed and approved the study protocol. All methods were carried out in accordance with relevant guidelines and regulations.

### Experimental Paradigm

During MEG recording, participants sat in a nonmagnetic chair within a magnetically-shielded room and performed a visual SWM task (Fig. 1). Participants were instructed to fixate on a centrally-presented crosshair that was embedded within a 7 × 9 grid throughout the task. Each trial began with the presentation of the crosshair and an empty grid for 1500 ms. Next, four black squares were presented within the grid, one in each quadrant (i.e., encoding). After 1500 ms, these black squares disappeared and the empty grid remained on the screen for 2500 ms (i.e., maintenance). Subsequently, a probe consisting of four black squares was presented for 1000 ms (i.e., retrieval). In 50% of trials, the probe was identical to the previous encoding stimulus, while in the remaining trials the probe differed in that one of the previously presented black squares had moved in location by one square within the grid. Additionally, the crosshair changed from blue to green during retrieval, to cue participants to respond. Participants were instructed to respond via button press as to whether the probe was identical to the previous encoding stimulus (yes or no). Each trial lasted 6500 ms and there were a total of 128 trials, resulting in a total run-time of ~14 minutes.

### MEG data acquisition

Recordings occurred in a one-layer magnetically-shielded room with active shielding engaged. Neuromagnetic responses were sampled continuously at 1 kHz, with an acquisition bandwidth of 0.1–330 Hz, using an Elekta MEG system featuring 102 magnetometer and 204 planar gradiometer sensors (Elekta, Helsinki, Finland). MEG data from each participant were individually corrected for head motion and noise reduced using the signal space separation method with a temporal extension^100,101^.

### MEG Coregistration & Structural MRI Acquisition and Processing

Preceding MEG measurement, four coils were attached to the participant’s head and localized, together with the three fiducial points and scalp surface, with a 3-D digitizer (Fastrak 3SF0002, Polhemus Navigator Sciences, Colchester, VT, USA). During MEG recording, an electric current with a unique frequency label (e.g., 322 Hz) was fed to each of the coils, inducing a measurable magnetic field that allowed each coil to be localized in reference to the sensors throughout the recording session. Since coil locations were also known in head coordinates, all MEG measurements could be transformed into a common coordinate system. With this coordinate system, each participant’s MEG data were coregistered with structural T1-weighted neuroanatomical data before source space analyses using BESA MRI (Version 2.0; BESA GmbH, Gräfelfing, Germany). These data were acquired with a Philips Achieva 3T X-series scanner using an eight-channel head coil (TR: 8.09 ms; TE: 3.7 ms; field of view: 240 mm; slice thickness: 1 mm; no gap; in-plane resolution: 1.0 × 1.0 mm). Structural MRI data were aligned parallel to the anterior and posterior commissures and transformed into standardized space, along with the functional images, after beamforming (see *MEG Source Imaging & Statistics*).

### MEG Time-Frequency Transformation and Statistics

Cardiac-artifacts were removed from the data using signal-space projection (SSP), which was accounted for during source reconstruction^102^. The continuous magnetic time series was divided into epochs of 5500 ms duration, with the onset of the encoding stimulus being defined as 0 ms and the baseline being defined as the 400 ms preceding encoding (i.e., −400 to 0 ms). Given our task and epoch design, maintenance onset occurred at 1500 ms and retrieval onset occurred at 4000 ms. Epochs contaminated with artifacts were rejected based on a fixed threshold method, supplemented with visual inspection. Briefly, for each individual, the distribution of amplitude and gradient values was approximated across all trials, and those trials containing the highest amplitude and/or gradient values relative to the full distribution were rejected by selecting a threshold that excluded extreme values. Importantly, these thresholds were set individually for each participant, as inter-individual differences in variables such as head size and proximity to the sensors strongly affects MEG signal amplitude because these variables affect the distance between neuro-electric currents and the MEG sensors. Additionally, only correct trials were included in subsequent analyses. On average, 82 (range: 66–98) correct trials per participant remained after artifact rejection. Artifact-free epochs were transformed into the time-frequency domain using complex demodulation with a resolution of 1.0 Hz and 50 ms from 4 to 50 Hz. This procedure involves filtering the complex signal into a number of frequency bands of a predetermined width and overall range (e.g., 1 Hz bands from 4 to 50 Hz), and calculating the power within each band across each successive temporal window^103,104^. Of note, this time-frequency decomposition method has been employed in a wide variety of previous neurophysiological studies^71,79,105–110^. The resulting spectral power estimations per sensor were averaged across all trials to generate time-frequency plots of mean spectral density. These sensor-level data were then normalized using the −400 to 0 ms time period.

The time-frequency windows used for imaging were determined by statistical analysis of the sensor-level spectrograms across all gradiometers during the encoding and maintenance periods. Each data point in the spectrogram was initially evaluated using a mass univariate approach based on the general linear model (GLM). To reduce the risk of false positive results while maintaining reasonable sensitivity, a two-stage procedure was followed. In the first stage, one-sample *t*-tests were conducted on each data point and the output spectrogram of *t*-values was thresholded at *p* < 0.05 to identify time-frequency bins containing potentially significant oscillatory activity across all participants. In stage two, time-frequency bins that survived this threshold were clustered with temporally and/or spectrally neighboring bins that were also significant, and a cluster value was computed by summing the *t*-values of all data points in the cluster. Nonparametric permutation testing was then used to derive a distribution of cluster-values and the significance level of the observed clusters (from stage one) were tested directly using this distribution^111,112^. For each comparison, at least 10,000 permutations were computed to build a distribution of cluster values. Based on these analyses, only the time-frequency windows that contained significant oscillatory events across all participants were subjected to the beamforming (i.e., imaging) analysis. Thus, a data-driven approach was utilized for determining the time-frequency windows to be imaged.

### MEG Source Imaging & Statistics

Cortical networks were imaged through an extension of the linearly constrained minimum variance vector beamformer^113–115^, which calculates source power for the entire brain volume by employing spatial filters in the frequency domain. The single images were derived from the cross spectral densities of all combinations of MEG gradiometers averaged over the time-frequency range of interest, and the solution of the forward problem for each location on a grid specified by input voxel space. The source power in these images was normalized per participant using a separately averaged pre-stimulus noise period (i.e., baseline) of equal duration and bandwidth^114^. MEG pre-processing and imaging used the Brain Electrical Source Analysis (version 6.1) software.

Normalized source power was computed for the selected time-frequency bands over the entire brain volume per participant at 4.0 × 4.0 × 4.0 mm resolution. Each participant’s functional images were transformed into standardized space using the transform that was previously applied to the structural images and then spatially resampled (see *MEG Coregistration & Structural MRI Acquisition and Processing*). To assess the anatomical basis of the significant oscillatory responses identified through the sensor-level analysis, the resulting 3D maps of brain activity were statistically evaluated using a mass univariate approach based on the GLM. That is, one-sample t-tests were used to calculate statistical parametric maps per frequency band across all participants during the encoding and maintenance phases. The output statistical maps were displayed as a function of alpha level (*p* < 0.000001), and adjusted for multiple comparisons using a spatial extent threshold (i.e., cluster restriction, *k* = 300 contiguous voxels) based on the theory of Gaussian random fields^116–118^. To quantify the temporal dynamics, we extracted virtual sensors (i.e., voxel time series) from the peak voxel of each cluster in the statistical parametric maps. When computing the virtual sensors, we applied the sensor weighting matrix derived from the forward solution to the preprocessed signal vector, which yielded a time series for the specific coordinate. Additionally, to ensure that transient evoked responses did not account for the responses observed, we conducted the same virtual sensor time series analyses for each theta and alpha peak after removing evoked activity.

### Whole-Brain Performance Correlation Maps

To identify brain regions in which oscillatory activity was related to SWM performance, we utilized the data from the individual whole-brain maps computed in the previous step (see *MEG Source Imaging & Statistics*). We performed a Pearson correlation using each participant’s maps and their respective accuracy on the task. Specifically, we computed correlation maps between theta activity and accuracy, alpha activity and accuracy, and beta activity and accuracy. Reaction time was included as a covariate in these calculations, and as such, the final product was a whole-brain map of partial correlation coefficients, per time-frequency bin of interest. These correlation maps were displayed as a function of alpha level, and adjusted for multiple comparisons using a cluster restriction (*k* = 300 contiguous voxels).

### Data availability

The datasets generated during and/or analyzed during the current study are available from the corresponding author on request.

## Electronic supplementary material

Supplementary Information
