# Supplementary material for: Beta Oscillatory Dynamics in the Prefrontal and Superior Temporal Cortices Predict Spatial Working Memory Performance
Source: Sci Rep. 2018 May 31;8:8488. doi: 10.1038/s41598-018-26863-x (PMC5981644; doi:10.1038/s41598-018-26863-x)
Supplement: Supplementary file 1 — Supplementary Information [file 41598_2018_26863_MOESM1_ESM.pdf]

Beta Oscillatory Dynamics in the Prefrontal and Superior Temporal Cortices Predict Spatial Working Memory Performance

Amy L. Proskovec[a,b,c], Alex I. Wiesman[b,c], Elizabeth Heinrichs-Graham[b,c], & Tony W. Wilson[a,b,c,CA]

[a] Department of Psychology, University of Nebraska - Omaha, NE, U.S.A.
[b] Center for Magnetoencephalography, University of Nebraska Medical Center (UNMC), Omaha, NE, U.S.A.
[c] Department of Neurological Sciences, UNMC, Omaha, NE, U.S.A.

Corresponding Author:
Tony W. Wilson, Ph.D.
Center for Magnetoencephalography
University of Nebraska Medical Center
988422 Nebraska Medical Center
Omaha, NE 68198
Phone: (402) 552-6431
Fax: (402) 559-5747
Email: twwilson@unmc.edu

Supplementary Information

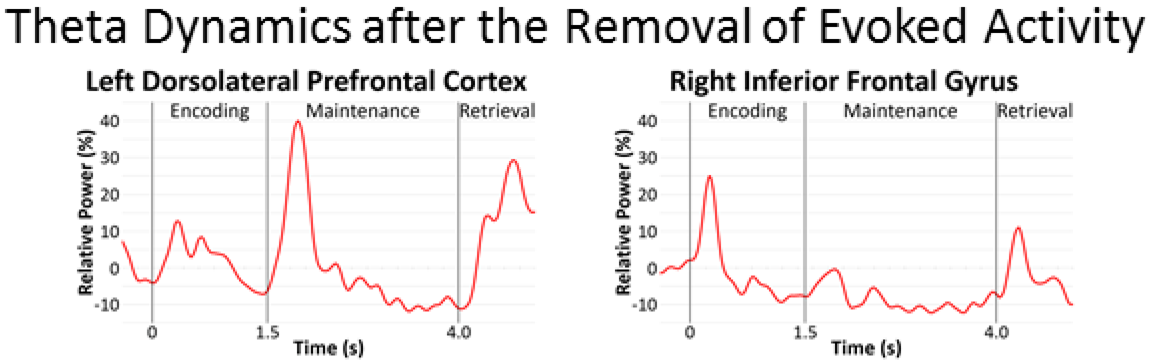

Supplementary Figure S1. Time courses of theta activity from the peak voxel in the left dorsolateral prefrontal cortex (DLPFC) cluster and right inferior frontal gyrus (IFG) cluster (shown in Figure 3) after the removal of evoked activity. Each region revealed a transient increase in theta activity following the onset of the encoding grid, with a more substantial increase seen in the right IFG (right). A transient increase in theta activity was also found at the beginning of the maintenance phase in the left DLPFC (left).

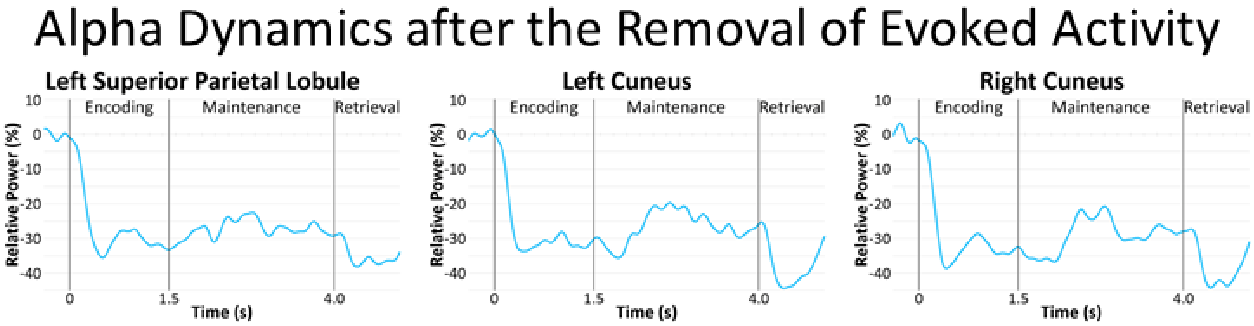

Supplementary Figure S2. Time courses of alpha activity from the peak voxel in the left superior parietal lobule cluster, left cuneus cluster, and right cuneus cluster (shown in Figure 4) after the removal of evoked activity. The alpha time series from the peak voxel of each region revealed strong decreases following the onset of the encoding stimulus across all three regions, and these decreases persisted throughout the remainder of encoding and maintenance processes.
